# Family-Based Consent and Motivation for Cadaveric Organ Donation in China: An Ethical Exploration^1^

**DOI:** 10.1093/jmp/jhz022

**Published:** 2019-09-16

**Authors:** Ruiping Fan, Mingxu Wang

**Affiliations:** 1 City University of Hong Kong, Hong Kong, PRC; 2 Xi’an Jiaotong University, Xi’an, PRC

**Keywords:** *Confucian family-based ethics*, *filial piety*, *opt-in*, *opt-out*, *organ donation and motivation*

## Abstract

This essay indicates that Confucian family-based ethics is by no means a stumbling block to organ donation in China. We contend that China should not change to an opt-out consent system in order to enhance donation because a “hard” opt-out system is unethical, and a “soft” opt-out system is unhelpful. We argue that the recently-introduced familist model of motivation for organ donation in mainland China can provide a proper incentive for donation. This model, and the family priority right that this model supports, is ethically justifiable in terms of Confucian family-based ethics.

## I. INTRODUCTION

Studies have shown that cadaveric organ donation is both medically beneficial and economically desirable. In particular, significantly lower mortality, better quality of life, and doubled life expectancy are associated with renal transplant recipients (Tonelli et al., 2011). Compared to dialysis, kidney transplantation can also lead to the substantial reduction of health-care costs (University of Maryland Medical Center, 1999). Against this background, if cadaveric organ donation is voluntarily initiated out of the moral motive of saving human life and is properly operated without violating any significant medical or moral norms, such as failures to follow appropriate death criteria or requirements for valid informed consent, it would be ethically admirable for individuals to donate their organs after death, because such donations bring about medically and economically better consequences. However, the shortage of organs for transplants remains a global problem. The demand for organs (especially kidneys) in China far outstrips the supply.

Although donation rates have gradually risen in recent years, it is estimated that only about 10,000 donated organs are available every year in China, whereas at least 300,000 patients are waiting for organ transplantations (Pan, 2013; Xinhua News Agency, 2015; CIIC, 2016). This means that only one out of 30 patients on the waiting list are likely to obtain an organ for transplantation. Thus, it is necessary to investigate what factors have prevented the Chinese from becoming deceased donors and explore ethically acceptable and practically effective ways to increase cadaveric organ donations in China.

China started its organ transplantation program in the 1960s and has experienced complicated changes in regulations and reviews in recent years (Ding, 2006, 2008). For years, China was the only country systematically to use organs from executed prisoners in transplantation procedures: 65 percent of the transplants done in China used organs from deceased donors, over 90 percent of whom were executed prisoners (Huang et al., 2012, 862). Under Western pressure, in late 2014 China’s national organ donation and transplantation committee required that all Chinese hospitals stop using organs from executed prisoners and that civilian organ donation would be the sole source for organ transplant in China, starting January 2015 (BBC News, 2014).^2^ However, the goal of increasing civilian donation rates cannot be achieved by abolishing donations from executed prisoners alone. Altruism needs cultural motivation.

A frequently heard critique is that Confucian family-based ethics, which is still vibrant in contemporary China, constitutes a stumbling block to organ donation. We deal with this critique in section II. We argue that it is categorically misguided to blame Confucian family-based ethics for the low rate of organ donation in China. What is needed, to the contrary, is to discover proper and effective ways to optimize organ donation by drawing on the mainstream Confucian cultural forces that are influential in the country. Specifically, the second section of this essay shows why Confucian family-based ethics supports deceased donation in principle and could be drawn on to encourage donation in practice. In the third section, we contend that China should not change to an opt-out approach in order to enhance donation, because such an approach is seriously ethically dubious and practically ineffective. Then, in section IV, we argue that the familist model of motivation for organ donation, as it is structured in China, will not only help optimize the supply of organs for transplantation, but will also be ethically justified. The final section covers concluding remarks.

## II. IS CONFUCIAN FAMILY-BASED ETHICS A STUMBLING BLOCK TO ORGAN DONATION?

As a number of Chinese bioethicists have argued, a long-standing Confucian family-based ethical tradition is still vital in Chinese societies and should be drawn on in formulating relevant health-care policies and directing Chinese bioethical practices (Li, 1997; Fan, 1999; Tao, 2004; Qiu, 2004; Lee, 2007; Fan, 2010a; Nie, 2015; Yung, 2015; Zang, 2017). This ethical tradition is embodied in a familist way of life: immediate family members share important life decisions with each other and make family decisions for each other, including health-care choices as well as living and deceased organ donation decisions (Fan, 2015). As a sociological fact of the matter, no one would doubt that a significantly important cultural role has been played by this Confucian family-based ethics in various dimensions of Chinese lives. But, there are controversies regarding what normative ethical implications this ethics has had on organ donation issues. Those who are critical of Confucian ethics in relation to the shortage of organ donations in China raise two charges. The first is that, given that Confucian family-based ethics is a major cultural force in directing Chinese lives and practices, it is hard to believe that it has nothing to do with the low rate of organ donation in China (Chen, 2013). The second charge pertains to the Confucian virtue of filial piety (*xiao*), which requires that one should not damage any part of the body in order to show proper filial piety to one’s parents and ancestors. This virtue, according to critics, discourages the Chinese people from donating their organs after death, because if an individual were to donate organs, he would not keep his body intact (Bo, Li, and Wang, 2005; Zhang, Hong, and Bai, 2009).

To deal with the first charge, we need to consider the sharp contrast between the high approval rate for donation in social polls and the actual low rate of registration for donation (e.g., Yan, Huang, and Qiu, 2015). Many polls show that about 20 percent of Chinese adults are willing to donate their organs after death, but very few have actually registered to become deceased donors. We think this contrast suggests a few things. First, given that many people are willing to donate organs after death, and given that Confucian family-based ethics is the main cultural force of the society in affecting people’s conduct, then it could hardly be true that such a positive attitude toward donation was shaped only through rejecting Confucianism. To affirm their charge, critics need to show that the high rate of willingness to donate is not from the influence of Confucian ethics, while the low rate of registration or donation is indeed generated by the impact of Confucian ethics. We have seen no evidence for either of these possibilities. On the other hand, it is evident that donation rates are multi-causal in every society. In addition to cultural factors, economic incentives and institutional arrangements have an important influence on donation rates. Accordingly, to understand why donation rates are so low in China, one will need to look not only at cultural values such as Confucian ethics, but also at Chinese economic circumstances and institutional settings that have been established for organ registration and donation in mainland China.

The Chinese system fails to offer adequate incentives, either honorary or financial, for organ donation. It provides no honorary incentives similar to those offered by the American Organ Procurement Organizations (OPOs) to donors and their families in the United States, such as a donor memorial garden, religious brochures on donation, the Donor Family Memorial box, the Donor Medal of Honor, special Gift of Hope flags, a sympathy card, a detailed outcome letter, a certificate of honor, a “We Remember” card, specialist services, and so on. They do all these things to gain the trust and confidence of potential donors and their families. They wish to emphasize that they are not solely interested in finding organs for recipients but are also committed to supporting donor families through their journey. Although the Chinese government has planned to provide financial social welfare compensation for needy families of deceased donors (Wu and Fang, 2013), our communication with relevant practitioners has revealed that such programs have become controversial and have not been effectively carried out. Some critics are worried that providing financial aid would change donor motivation from altruism to that of improving the economic interests of their families. Such a practice seems to critics as generating the risk of organ selling, which is taken to be immoral by some people, especially by many Western human rights groups that frequently put political pressure on the Chinese government with regard to organ donation policy.^3^

In addition, institutional arrangements to register as an organ donor are not user-friendly in China. For years the only available way of registering was to mark one’s desire to donate upon death due to automobile accidents when receiving a driver’s license. The Chinese, however, have a psychological disinclination for such “active” registration. They are afraid that this sort of consent will be self-fulfilling (*yi yu cheng chen*). So, it has been suggested that registration take place in the context of receiving one’s medical insurance card (Wang, Bai, and Yin, 2015). Others are worried that this change may not really improve psychological effects, but that it may generate other problems, such as reducing public trust in the integrity of insurance schemes and medical institutions. All these complexities indicate that it is hard to conclude that it is the influence of Confucian ethics, rather than the failure to provide proper incentives or a user-friendly registration system that is accountable for low donation rates in China.

The second charge regards how the Confucian virtue of filial piety should be appropriately interpreted. It is true that filial piety is a fundamental virtue in Confucian family-based ethics, and it is taken to be the root of the very Confucian virtue *ren* (humanity or benevolence) in the tradition. It is also true that the Confucian classic *Of Filial Piety*, explicitly states that one’s body, skin and even hair are all from one’s parents so that to be a filial person one should not damage any of them. Critics infer that this statement indicates that Confucianism does not and cannot support organ donation because it would damage one’s body. However, this inference is obviously inconsistent with the basic teaching of Confucian virtue ethics: one should cultivate oneself to become a virtuous person in loving and taking care of other people, even at the risk of one’s life if necessary. To be consistent with this basic teaching, there has long been an implicit distinction within Confucian tradition between legitimate and illegitimate risk-taking, including risks that involve the possibility of damaging part of one’s body, as numerous historical examples and cases illustrate. Thus, *Of Filial Piety* ought to be understood as arguing that illegitimate, but not all, body-risk-taking acts against filial piety. Given that organ donation is made for loving and assisting other people, Confucianism would not, and cannot, define it as a type of illegitimate body-risk-taking. Indeed, Confucius in the *Analects* advocates that “the man of *ren* is one who, desiring to sustain himself, sustains others” (Confucius, 1992; Analects 6.30). It is clear that the Confucian idea of filial piety cannot be interpreted as objecting to the modern notion of organ donation because transplantation is designed to contribute part of one’s body to save human life. In fact, some Chinese scholars have indicated that the notion of “damage” used in the original text *Of Filial Piety* is actually a notion of “damage via criminal penalty” rather than a general kind of “damage” (Chen, 2013; Fang, 2014). The idea is that a filial person should not perform improper or criminal conduct, which might result in corporal punishment on one’s body by the law and thus violate the virtue of filial piety.^4^

In contemporary China, sometimes relatives, especially adult children, refuse to carry out the patient’s wish to donate organs after death, because they fear that agreeing to such a wish would not be filial (*bu xiao*) to their parents because it would fail to keep the body intact. However, as some scholars have pointed out, this is a misunderstanding of the Confucian requirement of filial piety. Since organ donation is an act of *ren*, honoring one’s parents’ wish for donation after death is following the Confucian teaching of loving others, and a virtuous person does not always need to keep the body intact. Moreover, it has been demonstrated that after cadaveric donation, necessary Confucian rituals, such as those for funeral, sacrificial, and ancestor reverence, could all be appropriately performed in practice without improper interference (Fang, 2015). In fact, more and more adult children have come to embrace this legitimate Confucian understanding of filial piety and are willing to follow their parents’ decisions for deceased donation.

In short, when critics attempt to blame Confucian family-based ethics as a stumbling block to organ donation, it is both theoretically ill-informed (because it is not the genuine meaning of Confucian filial piety) and practically counterproductive (because fewer and fewer children go against their parents’ wishes to donate). What we need to note is that actual organ donation rates are affected by many variables, including cultural, economic, and institutional factors. China needs to find a proper and effective way of optimizing organ donation by drawing on mainstream Confucian cultural forces in the country. We will return to this issue in the following sections.

## III. A HARD OPT-OUT SYSTEM IS UNETHICAL, AND A SOFT ONE IS UNHELPFUL

Chinese families under the Confucian family-based ethics are not the only socio-cultural group that holds that they should have the right (as well as the authority) to reconfirm or to refuse organ donation on behalf of a deceased family member who expressed consent before death. In those countries that require “explicit individual consent” to donate, their families are also very often involved. Even in countries (such as France) that have adopted so-called “presumed consent” to donation, families may reject donation from a deceased member. In the United States, organ retrieval teams routinely seek family authorization to obtain organs when the soon-to-be-deceased potential donor has not given prior consent. In such cases, the family is asked to donate the patient’s organs upon his or her death. Some jurisdictions have begun using Rapid Organ Recovery (ROR) protocols. Following an unexpected death, such as cardiac arrest or severe trauma resulting in significant blood loss, organ preservation is begun at once, even prior to determining the patient’s wishes and before family members are present to give consent. Indeed, in the United States, the United Network for Organ Sharing has announced that it does not need the family’s informed consent in such cases; it has moved to requiring mere authorization. We believe that such practices are not only theoretically problematic but also practically dangerous because they may setback the overall interests of the donor. Such practices are likely to generate serious objections from family-oriented cultures, such as the Confucian Chinese. As Ana Iltis argues, this shift to mere authorization ought to be rejected, and we should recognize the ethical obligation to obtain valid informed consent (Iltis, 2015, 369). We agree with Iltis that “laws and practices should enable people easily to specify a role for their family in final decisions if they desire and to distinguish between a mere willingness to donate and a desire to be a donor given the implications of such a desire” (Iltis, 2015, 379).

Many seem to believe that replacing an opt-in system with an opt-out system would enhance organ donation. We take this conclusion to be misinformed. Both mainland China and Hong Kong are adopting opt-in systems—only those who have given explicit consent are donors, whereas some European countries are shifting to opt-out systems—anyone who has not clearly refused is a donor. When comparing the data of various countries, some studies show that opt-out systems lead to a relative increase in the total number of organs transplanted (e.g., Shepherd et al., 2014). However, even if changing to a “hard” opt-out system (in which organs are automatically taken regardless of families’ views or wishes, unless the person had explicitly objected during his lifetime) would increase organ donations, it would be unethical; moreover, adopting a “soft” opt-out system (in which family members would be able to veto organ donation even if no formal objection had been made in the past by the deceased person) would be ineffective, even if it might be ethical.

First, almost all opt-out countries have in fact adopted some form of a “soft” rather than a “hard” approach. This is understandable, because it might be impossible to defend a “hard” opt-out system even for a modern Western individualist society, let alone for a Chinese familist society. On the one hand, in the West, individual autonomy or personal wishes must be respected to justify donation authorization. As an influential British ethical council points out, the importance to be attached to the person’s own wishes rules out absolutely any consideration of introducing a “hard” opt-out approach to deceased organ donation, given the impossibility of ensuring that everyone would be sufficiently well-informed to have the opportunity of opting out during their lifetime (Nuffield Council on Bioethics, 2014, 10). It is only reasonable to assume that families are in a better position to know the potential donor’s actual wishes. On the other hand, in the Confucian familist culture of China, it is generally held that the body, organs included, does not exclusively belong to an individual; rather, it belongs both to the individual and to his/her entire family, since the body is normally taken to be a gift that the individual received from his/her ancestors, especially his/her parents (Fan, 2010b; Wang and Wang, 2010). Accordingly, most Chinese think that it is ethically required that one’s immediate family members assist with medical decision-making and have a right to give consent or refusal regarding organ donation. Indeed, one is expected to consult with one’s immediate family members when deciding to become a donor and must obtain their consent before formally registering as a donor, or choosing to donate while living. Thus, it is only logical that one’s family must be sought for consent regarding organ donation at the time of one’s death, if one has not clearly expressed one’s wish during one’s lifetime. From the perspective of Confucian family-based ethics, this procedure is most reasonable not only because the family knows one’s wishes better than others, but also because it is morally mandatory that the family be engaged in the process of consenting to organ donation. Individuals are normally taken to be a member of the family, understood as an ethical unity, for critical decisions (Cai, 2015).

Moreover, there is a key issue of public trust that must be engaged. A “hard” opt-out strategy would inevitably bring about circumstances in which medical professionals would be intervening to “take” organs from individuals and their families rather than facilitating the organs being “given” by the individuals and their families. In such cases, a significant degree of trust in the medical system would be lost. Individuals and their families would very likely believe that the relevant government agencies and medical professionals are acting in collusion with each other to steal organs for the social “benefit” of organ transplantation, while deliberately excluding family involvement in the process to protect their members. This moral and social cost is too huge to pay.

A “hard” opt-out strategy aside, it is also unclear whether a “soft” opt-out strategy can be justified. The latter strategy for presumed consent seems permissible provided that sufficient opportunities are offered to individuals to register their objections and that their immediate family members have a right to veto organ harvesting if the individual has not already opted out. However, there are still serious moral concerns that such an opt-out system will compromise significant ethical values by failing to respect individual preferences or autonomy (AMA Council on Ethical and Judicial Affairs, 1994; MacKay, 2015). Opting out of the system is a viable option only for those patients who understand how the system works and know how to express their objections to donation (in China, a very large percentage of patients will not have such capacity). Moreover, there may be incentives under presumed consent to avoid discussions with patients and their families concerning organ donation for fear of discovering objections that would preclude retrieval. Concerns to avoid having patients opt-out might become in effect a reason for avoiding conversations about consent to organ donation entirely. Anyone considering recommending such a “soft” opt-out system for China must not simply ignore such concerns when attempting to formulate a new policy.

However, even if such a “soft” opt-out system could be ethically defended, we do not think it would work better than the current opt-in system for enhancing donation in mainland China or Hong Kong. Although we recognize that an opt-out system might bridge the gap between an individual’s intention and his/her behavior by removing the need to undertake any action in order to become a donor (Johnson and Goldstein, 2003), the reasons individuals donate are multi-factorial, and, as a result, an opt-out strategy may not actually be helpful. In fact, there is not much evidence that an opt-out approach will be particularly effective. For example, Spain has had the world’s best donation rate, but this has not been accomplished through its opt-out legislation, which was adopted in 1979. The legislation did not have a positive influence on donation for 10 years. Instead, crucial organizational changes have taken place (such as the introduction of incentives offered to its coordination networks and hospital coordinators) since 1989 that Spain has implemented successfully in recent years (e.g., Fabre, 2014). It has been publicly stated that the presumed consent law in Spain is essentially dormant (Fabre, Murphy, and Matesanz, 2010). In the case of mainland China or Hong Kong, even under their current opt-in system, it is the norm that immediate family members may decide in favor of deceased organ donation, if the person has not clearly expressed his own preferences. We can reasonably conjecture that simply replacing current opt-in practices with a “soft” opt-out system would not effectively change donation outcomes in either Hong Kong or mainland China. It would continue to remain up to families to make final decisions. In fact, international research has also demonstrated that next-of-kin have a considerable influence on the organ procurement process in both presumed and explicit consent nations (Rosenblum et al., 2012).

Accordingly, it is ethically proper for China to adopt explicit consent, rather than presumed consent, in organ donation decisions. In light of Confucian family-based ethics, we recommend that potential Chinese donors, who are considering donating their organs after death, first discuss their wishes with their immediate family members and gain their support. Unless the family has agreed, one should not formally register to be a cadaveric organ donor. Moreover, it is also ethically appropriate and necessary for the registration organization to secure the consent of the family, as well as the individual, through the formal signature of an immediate family member who serves as the representative of the family. Without the family’s signature, the desire of a potential deceased donor should be taken as invalid. This requirement could also serve as an effective mechanism to prevent family members’ objection to donation after the donor dies, since they have already agreed. Taking all of these considerations together, it is much more beneficial for us to find efficacious and defensible measures that are consistent with the Confucian family-based ethics to optimize donation in China than to move to an opt-out strategy.

Before we complete this section, we would like to say a word about the cultural legitimacy of those Chinese cases in which the family exercises a right to veto the potential deceased donors’ decisions to donate organs. Here, the situation of Hong Kong is heuristic. There exists a gap between legal ordinance, on one hand, and cultural practice, on the other. Hong Kong’s Medical (Therapy, Education, and Research) Ordinance (Chapter 278) stipulates that

If any person, either in writing at any time or orally in the presence of 2 or more witnesses during his last illness, has expressed a request that his body or any specified part of his body be used after his death for therapeutic purposes or for purposes of medical education or research-

(a) the person who has lawful possession of his body after his death may, unless he has reason to believe that the request was subsequently withdrawn, authorize in writing the removal from the body of any part or, as the case may be, of the specified part, for use in accordance with the request; and

(b) an authorization validly given under paragraph (a) shall remain valid notwithstanding any objection by the next of kin of the person who has expressed the request to the body being dealt with in accordance with the request after the death (Section 2).^5^

Here, 2(b) contains the words “notwithstanding any objection by the next of kin,” which seems to exclude a veto right held by the family. But, the reality of medical practice is different. No donation coordinator or medical professional in Hong Kong would go against a family’s final decision, whether for or against donation. Indeed, relevant regulative documents proposed by Hong Kong’s Legislative Council, Department of Health, and the Hong Kong Medical Association all require that a “family member of the deceased has to sign a consent form to confirm the organ or tissue to be removed before the transplant,”^6^ although this is not required by Medical Ordinance (Chapter 278) or the Human Organ Transplant Ordinance (Chapter 465). No one has offered any official explanation for the difference between law and practice in Hong Kong. Our conjecture is that the Ordinance was established following the relevant related British law, whereas the practice had to be adjusted to fit into the Chinese family-based ethical culture of Hong Kong, which means that a family’s veto right has been effectively established in practice. This right can be taken as consistent with the implication of part 2(a) of the Ordinance: “unless he has reason to believe that the request was subsequently withdrawn.” In Hong Kong, it is generally appreciated that compared to medical professionals or other relevant parties, the family is in a much better position to know whether the newly deceased request is still valid. In addition, the form used for organ donation registration in Hong Kong is very simple and fails to capture adequate information or to require many essential details.^7^ Accordingly, the record of organ donation registered by a person can only serve as a reference for the deceased’s wish and must be confirmed or denied by immediate family members in order to be morally valid.

Maintaining a family veto does not necessarily violate individual autonomy. If one has an impulse to register as a deceased donor through Hong Kong’s internet-based system, one has not been adequately informed of relevant issues. One does not truly understand what one is actually consenting, such as under what circumstances and by which death criterion organs will be removed, nor is there a medical professional available to answer questions. Due to the lack of such necessary information, an internet registry is hardly evidence of an autonomous decision. In a Confucian-influenced family-based culture like Hong Kong, the family is normally appreciated as improving an individual’s capacity to exercise autonomy (Fan and Chan, 2017). Consequently, the family veto ought to be kept. In addition, we recommend that registration forms should be designed to provide as sufficient information as possible and should also require the signature of a family representative to secure valid informed consent.

In short, to improve donation rates in mainland China or Hong Kong, we should not attack Confucian family-based ethics or shift to an opt-out strategy. Instead, we should attempt to draw on Confucian ethics to propose effective measures that are ethically defensible. Our recommendation for a family signature as part of registration to become an organ donor would be an important step in the right direction.

## IV. THE FAMILIST MODEL OF MOTIVATION FOR DONATION IS JUSTIFIABLE

Chinese and international transplant researchers and practitioners have recognized that organ donation and transplant systems inevitably need to work within the characteristics of the local cultural and socioeconomic context. Even in the United States, we see regional variations in practice, although not so much in law. In some places, organ procurement organizations are willing to take organs against a family’s wishes whereas in other parts of the United States, they are not. As some transplant surgical professionals have pointed out, since China’s culture and stage of socioeconomic development is very different from the West, the dominant Western model of organ transplantation cannot be fully duplicated in Chinese society (Huang, 2007; Huang et al., 2015). Recently, the Chinese government and relevant social agencies have attempted to formulate culturally sensitive policies to develop organ donation and transplantation programs in China. Indeed, a document issued by the Chinese Ministry of Health titled “China’s Basic Principles of the Distributing and Sharing of Human Organs and the Core Policy for Liver and Kidney Transplantations” in late 2010 (Ministry of Health, 2010), assigned a priority right to organ donors and their immediate family members, if they should ever need transplantation. Specifically, the principle states that a living donor or the immediate family members of a cadaveric donor, whenever in need of liver or kidney transplantation, has a reasonable priority right to the distribution of donated organs. The document explains that this priority right has been established to encourage organ donation and to enhance the donors’ sacrificial spirit of saving others’ lives. This can be termed a Chinese familist model of motivation for organ donation, which establishes a family priority right. This model draws on the Confucian cultural value of differentiated and graded love in favor of immediate family members to incentivize donation (Fan, 2016a).

This familist model of motivation for organ donation and its account of family priority is supported by the Chinese people, including bioethicists (e.g., Xu and Han, 2011). In 2012, the China Organ Donation Administrative Center was established to normalize and promote organ donation in mainland China, and on April 2, 2014, the Center’s official website started to accept voluntary donation registration online.^8^ From the announcement of the website until August 31, 2017, there had already been 310,620 persons registered to donate organs after death; 13,285 deceased patients donated organs, with a total of 36,613 organs donated. Although donations have increased dramatically, compared with the demand the donation rates are still very low. Our preliminary investigation has discovered that a family priority right is generally supported and welcomed by the people involved in transplantation and is taken to be both fair and beneficial in mainland China. The familist model has indeed motivated the Chinese to donate organs. We are told by relevant practitioners that this priority right has been implemented in mainland China in various ways. For example, if a patient on the waiting list has an immediate family member who donated an organ in the past, this patient would enjoy an allocation privilege for an organ as long as the organ is medically suitable for him or her. In addition, with the establishment of this right, there were many cases in which family members who were not medically suitable donors for the patient, so a member would decide to donate an organ to an unrelated patient so that his relative would gain priority allocation for another suitable organ. Chinese medical institutions are willing to help patients and their families to realize this priority right (Wang, 2016).

Israel was the first country to shape a familist model of organ donation. In 2008, Israel’s Parliament passed the Organ Transplantation Law, which grants priority on waiting lists for transplants to candidates who are first-degree relatives of deceased organ donors or who previously registered as organ donors themselves. The Law was publicized toward the end of 2010 and fully adopted in 2012. Recent data demonstrate that the higher authorization rate in Israel in 2011–2015 compared with 1998–2010 was driven almost exclusively by the increased authorization rate for next-of-kin of unregistered persons (Stoler et al., 2016). Such a familial priority right has not yet been legislated by the People’s Congress, and there have not been public campaigns to promote the model in China like those in Israel. Such a priority right assigned to family members should encourage Chinese individuals to support cadaveric donations, since they remain living a family-based and family-oriented way of life within a Confucian familist culture (Fan, 2010a, 2015). If they know their donation will create a priority right for their family members to enjoy, many will be motivated to donate. This motivational strength is confirmed by our investigation in some Chinese institutions, although we do not yet have systematic statistics to support the conclusion.

It is no difficulty to support this familist model of motivation for organ donation in terms of Confucian family-based ethical resources. In a recent work, one of the two authors provided detailed analysis of the Confucian ideas that support Confucian conceptions of moral obligations with preference to family members:

(1) One has more moral obligation to take care of one’s family members (such as one’s parents, spouse, and children) than others in one’s local or religious community (such as neighbors, friends, and acquaintances);
(2) One has more moral obligation to take care of those in one’s local or religious community than other citizens in the state;
(3) One has more moral obligation to take care of one’s fellow citizens in the state than other people in other states.^9^

The tenability of this Confucian moral system of differentiated and graded love and obligations requires us to object to any radical egalitarian requirement of health-care services in general or organ donation and allocation systems in particular. For example, if the state requires that everyone within any local community equally get access to or enjoy similar health services or goods (namely, there are policies and arrangements imposed in the state to make sure that no one gets better basic health-care services), this would violate the Confucian conviction that one has more moral obligation to take care of one’s family members than others within one’s local community or society. Similarly, if the state demands that no organ donor, by one’s voluntary and good-minded action of donation, should bring about any positive effect to the extent that certain preferential treatment of one’s family members in a similar medical context would take place, this would categorically contradict Confucian moral sentiments and requirements for differentiated and graded love in favor of one’s family. In short, the familist model of motivation for organ donation can be justified in light of Confucian moral resources of differentiated and graded love.

Scholars who promote a global bioethics would challenge us to offer general ethical justification for the model, independent of Confucian ethical culture. The legitimacy of the familist model, they might argue, should be assessed in terms of general moral precepts beyond Confucianism, such as the four bioethical principles constructed by Beauchamp and Childress: respect for personal autonomy, nonmaleficence, beneficence, and justice (Beauchamp and Childress, 2013). Of course, we have noticed that these four principles are frequently appealed to in contemporary bioethical accounts. However, what these so-called middle-level principles have offered is at best only a framework of general moral norms or values for bioethical approaches. Unless such general norms or values are further specified or interpreted in concrete situations, they cannot be applied to any particular bioethical position, such as the familist model of motivation under discussion. However, one can hardly provide such specification or interpretation independent of the major ethical starting points or fundamental premises of a particular ethical culture, because there would be no substantive moral resources to do the work. Accordingly, the best ethical argument we can offer for such a family priority right in terms of the four principles would be a more or less Confucian account of the four general norms or values regarding organ donation issues based on fundamental Confucian ethical ideas and fabrics.^10^

First, would Chinese individual action in exercising such a family priority right be non-autonomous in any serious sense? Beauchamp and Childress set three formal conditions for autonomous individual action: (1) intentionally, (2) with understanding, and (3) without controlling influences that determine their action (Beauchamp and Childress, 2013, 104). Here, the third condition might be the only one at stake: when an individual is motivated by the family priority right to decide to become a potential deceased donor (where he would not have made this decision absent such an incentive), is the establishment of such a right unduly controlling his decision? We think the Confucian answer is no. First, since the root of the influence of this right is normally a kind of emotional appeal (loving one’s family members), it is legally legitimate for one to resist the emotional appeal, so there is no threat of force involved in such a case. Moreover, the level of “controlling,” if any, is not nearly as strong as in the case of a living donation to save an ill family member. In the case of deceased donation, one is not confronted with a desperately needy family member who may die. If living donation can be understood as autonomous, then deceased donation, even with the incentive of a family priority right, must be more so. Finally, this influence can reasonably be understood as a kind of persuasion rather than coercion in the context of Confucian family-based ethics, because it is engaged with the normative Confucian moral reasons of loving and taking care of one’s family members, rather than forcing them to do anything.

The general norm of nonmaleficence requires one to abstain from causing harm to others (Beauchamp and Childress, 2013, 150). When one is motivated to donate one’s future cadaveric organs under the familist model, one certainly has *no intention* to inflict harm or evil on anyone, although one wants to provide special favor for one’s family members in the future. Now, is anyone actually harmed by the establishment of the family priority right even if there is no intention of harm? Suppose both patients A and B are similarly medically fit for an available organ, but while A’s position in the waiting list is medically before B, B receives the transplant because of the family priority right, since B has a family member who was a deceased donor. It seems that A would have obtained the organ absent this family priority right. In this case, has A been harmed by the establishment of such a right? No. The reason is that absent this right, the extra organ donated through the motivation of the familist model would not have been made available. If the organ had not been donated, while B would not have had a priority right to an organ, A would likely not have secured an organ either, because there would be significantly fewer organs available for transplant. Accordingly, under the family priority right, there is neither intention nor actuality of harm inflicted on any patient.

Beauchamp and Childress identify the moral norm of beneficence in two forms: positive beneficence and utility: “*Positive beneficence* requires agents to provide benefits to others. *Utility* requires that agents balance benefits, risks, and costs to produce the best overall results” (Beauchamp and Childress, 2013, 202; italics original). It is not difficult to support the family priority right in terms of both of these two forms in the Confucian context of China. The increase of donated organs through the establishment of such a right would save more patients’ lives, improve their quality of life, and enhance their life expectancy. It is also evident that the best overall results will be achieved for all persons involved with the familist model, provided that valid consent is secured from both the donor and the family based on sufficient relevant information being offered to them before they give consent.

Finally, the norm of justice is most complicated and deeply theory-laden. Beauchamp and Childress summarize six divergent theories and material principles of justice (2013, 253). They claim that “no single theory of justice or system of distributing health care is sufficient for constructive reflection on health policy” (2013, 293). We do not think their integrated strategy for health-care justice is either theoretically successful or practically feasible because such divergent theories and principles contain mutually incommensurable moral convictions resistant to integration. For example, we have to concede that the familist model and the family priority right cannot be supported by a radical egalitarian account of justice. Such a perspective tends to require us to weigh needs or interests impartially, taking no account of whose interests they are or of what relations others hold to us, contrary to basic Confucian moral beliefs and sensibilities. Thus, our reflections on the justice of the familist model and the family priority right cannot be made independently of Confucian moral and intellectual resources, especially their insight into the tenability of a relation-relevant morality regarding our obligations of providing care and assistance to others. From the Confucian point of view, radical egalitarianism is unreasonably demanding and cannot be defended. Based on the Confucian nonegalitarian justice of graded and differentiated love/obligation, it would be unfair and unjust for the government to leave no room for individuals to practice nonegalitarian love/obligation in favor of their family members. A requirement that prohibits any family priority in organ donation and distribution would be an unfair and unjust policy. Instead, the familist model is the most reasonable and just model of legitimate motivation for enhancing the supply of organs from deceased donors because it conforms to comprehensive Confucian moral convictions (Fan, 2016a). The credibility of this claim will eventually depend on the persuasiveness of Confucian justification for its family-based and family-oriented ethics as well as on the quality of how Confucian arguments respond to their critics.^11^

## V. CONCLUDING REMARKS

There are three types of incentives for improving organ donation: honorary, financial, and familist. Honorary incentives are the least controversial but also the least motivating, whereas financial incentives are the most motivating but also the most controversial (Kass, 1992; Krauthammer, 1999; Delmonico and Scheper-Hughes, 2002; Cherry, 2005, Bagheri, 2006; Mahdavi-Mazdeh, 2012; Siraj, 2016). Familist incentives, like the family priority right provided in the Chinese model, are a type of nonfinancial incentive that would be highly motivating at least within a family-based ethical culture like that of China. This familist model can be defended in light of substantive Confucian moral resources. As we have argued, Confucian family-based ethics is by no means a stumbling block to organ donation. China should not change to an opt-out consent system in the hopes of enhancing donation. Rather, the familist model should be employed to provide proper incentive measures that can promote donation while also being ethically justifiable. What is needed in China is to specify the standards of the model, to improve its implementation, and to conduct public campaigns in support. In short, China will need to draw on its cultural and ethical resources to develop suitable bioethical programs in general and organ donation programs in particular (Engelhardt, 1996; Fan, 2010a). To enhance cadaveric organ donation in China, family-based consent and motivation are both ethically defensible and practically effective. A family priority right under the familist model should be publicly respected and fully implemented.

## Footnotes

2. Some international scholars still cast doubt on the seriousness of China’s decision to stop using organs harvested from executed prisoners (Jha, 2015). Our limited investigation has found no more such use in China, although a recent article in the *New York Times* has suggested otherwise (Tatlow, 2015). However, it remains ethically controversial whether a prisoner who is to be executed should be allowed to donate organs. Interested readers should look at a useful article published in the *Journal of Medicine and Philosophy* by Wang and Wang (2010). Wang and Wang argue that organ donation from capital prisoners is ethically defensible if certain conditions are satisfied. The authors contend that the West should respect the tradition of Chinese families as vital life communities and that when vital issues are to be dealt with, family members are usually not anxious to publicize issues but will first discuss them among the family members to put forward a solution. Before the court and the hospitals allow capital prisoners to donate their organs, they should obtain the concerted consent of both the prisoners and their family members. If one party does not agree to donate organs, organ donation should not be realized. These considerations will ensure that organ donation will be based on the intention of caring for others and will be conducted with appropriate familial authority so as to eliminate pointless interference from authorities and ensure that donation is done in an ethical fashion (Wang and Wang, 2010, 207). From the information and argument provided by this article, we do not think that the fact that China’s recent organ donation program relied heavily on organs donated from executed prisoners should create a stigma around organ donation that might lead people to refuse to donate their organs, because well-informed, validly-consented-to and properly operated organ donations, even from executed prisoners, are taken to be ethically acceptable and even admirable in China (Wang and Wang, 2010, 209).

3. We do not think it is always morally wrong to offer financial aid to donors’ families, although we do not have space to address this issue in this essay. For general moral debates about organ sale, see Cherry (2005).

4. In ancient China, criminal penalties involved several types of corporal punishment.

5. Medical Ordinance: available at https://www.bing.com/search?q=Medical+Ordinance+%28Chapter+278%29&qs=n&form=QBRE&sp=-1&pq=medical+ordinance+%28chapter+278%29&sc=0–31&sk=&cvid=061C9CD86DE940BF91C42D309FC82FA6 (accessed in January 2018).

6. See, for example, Hong Kong Legislative Council document CB(2)836/15–16(08), available at http://www.legco.gov.hk/yr15-16/chinese/panels/hs/papers/hs20160418cb2-836-8-c.pdf (accessed in January 2018).

7. See Centralized Organ Donation Register, available at https://www.codr.gov.hk/codr/InternetAgreeRegistration.jsf (accessed in January 2018).

8. See China Organ Donation Administration Center: http://www.china-organdonation.org.

9. For detailed Confucian reasons and arguments for these graded and differentiated obligations, see Fan (2016b).

10. Mainstream bioethical discourses, in adopting the four principles, do not provide Confucian specifications or interpretations. This fact, however, does not mean that their specifications or interpretations are from nowhere. Given that they have to have their own ethical starting points or fundamental premises to do the work, theirs are, like the Confucian we offer here, also from particular ethical cultures, mostly liberal ethical culture.

11. In Fan (2016b), the author provides a series of considerations to defend Confucian nonegalitarian family-based and family-oriented ethics, including the impracticality or self-contradiction of radical egalitarian morality, support from discoveries of moral psychology, moral arguments around equality and inequality, and reflections on moral, political, and economic consequences generated by different moral views.
